# Supplementary material for: Beyond the Digital Competencies of Medical Students: Concerns over Integrating Data Science Basics into the Medical Curriculum
Source: Int J Environ Res Public Health. 2022 Nov 30;19(23):15958. doi: 10.3390/ijerph192315958 (PMC9739359; doi:10.3390/ijerph192315958)
Supplement: Supplementary file 1 [file ijerph-19-15958-s001.zip › ICTbioStats-SupplementaryFile1.pdf]

Syllabus : course of biomedical informatics (2017 – 2018)

1st academic year, 4 ECTS credits

14 weeks

General objective: knowledge and understanding of basic concepts in biomedical information technology and communication, with the specific methods and their adequate application in the biomedical field.

1. Introduction: medical informatics as an interdisciplinary science; course objectives and outline.

2. Medical information. Data *vs.* information. Information systems. Structured data. Data files.

3. Databases and database management systems. Data portability. SQL and NoSQL models.

4. Information systems. Information security and authentication methods. Symmetric and asymmetric cryptography. Digital signature.

5. Ethics issues in biomedical data handling. Security and confidentiality of medical data. Possible solutions in medical information systems.

6. Complex data structures: biosignals. Principles of sampling and quantization. Basics of digital biosignal processing, with ECG and EEG examples. Practical exercises with *LabChart Reader*.

7. Complex data structures: biomedical images and their digital representation. Digital images' sampling and quantization. Basics of image processing with examples from digital filtering, histogram-based analysis and segmentation. Practical exercises with *ImageJ*.

8. Computational biology and bioinformatics: basic concepts and their integration into the larger context of biomedical informatics.

9. Bioinformatics: biological databases and dedicated software tools. Projects of computational biology and bioinformatics towards the genomic medicine.

10. Bioinformatics web-based services. Examples of NCBI and EMBL services.

11. *eHealth* solutions for optimizing the medical processes. Health medical records and telemedicine. Basic concepts of *mHealth*: mobility, portability, and interoperability.

12. Opportunities and barriers in *mHealth*. Current practice examples and applications.

13. *mHealth* applications to facilitate the medical research and secondary use of medical data. The concept of big data and its health applications.

14. Biomedical information and informatics. Discussions, conclusions.

Syllabus: course of biostatistics (2018 – 2019)

1st academic year, 2 ECTS credits

14 weeks

General objective: knowledge and understanding of basic concepts in biostatistics and the applied quantitative methods in biomedical data processing.

1. Introduction in biostatistics: variable, population, sample.

2. Data description. Expected value, variance and standard deviation. Median and quartiles. Mode. Coefficient of variation.

3. Distributions. Normal distribution. Standard normal distribution.

4 – 5. Statistical estimation. Confidence intervals. One-sided and two-sided confidence intervals. Estimation of mean and proportion.

6 – 7. Statistical testing: concept, decision, errors. Common tests: z-test, t tests, Chi-square and ANOVA. One-sided and two-sided testing.

8. Statistical testing. Choosing the appropriate test. Principles for calculating the power of a test and sample-size determination.

9. Correlation and regression analysis. Examples with linear regression.

10. Biostatistics in epidemiology: observational studies, risk analysis.

11 – 12. Statistical methods in medical decision. Bayesian analysis. Evaluating performance of a diagnostic test: sensitivity, specificity, predictive values, ROC curve.

13 – 14. Clinical trials. Categories of research design; specific principles for collecting and analyzing medical data. Planning the statistical analysis; data format and management; reporting the results. International regulations.
