# Supplementary material for: Beyond the Digital Competencies of Medical Students: Concerns over Integrating Data Science Basics into the Medical Curriculum
Source: Int J Environ Res Public Health. 2022 Nov 30;19(23):15958. doi: 10.3390/ijerph192315958 (PMC9739359; doi:10.3390/ijerph192315958)
Supplement: Supplementary file 1 [file ijerph-19-15958-s001.zip › ICTbioStats-SupplementaryFile2.pdf]

TOOLS FOR DATA COLLECTION

Depression was measured with the nine-item Patient Health Questionnaire (PHQ-9), validated for the Romanian population. PHQ-9 is a short screening instrument initially used for detection of depression in primary care patients but proven to be a valid screening tool for depression in various settings and also for the general population. On PHQ-9, each of the nine items is subjectively scored on a scale from 0 ("not at all") to 3 ("nearly every day"). The total possible scoring ranges from 0 to 27 points (values over 14 signal a trend toward severe depression).

Seven questions probed students' perception of work effectiveness and overall satisfaction with life, health, on-line professional activity, and support from the University. Two questions collected their declared levels of on-line attendance during the spring and the ongoing semester. The face validity of these questions (namely their relevance to this project) was assured by a Delphi technique in the development process; no prior formal validation was conducted.

Residual knowledge of BMI and biostatistics was assessed with 14 multiple choice questions, with one or multiple correct answers. Questions' face validity was assured by a Delphi technique in the development process and the answers were manually coded by two independent reviewers, as it follows: 0 = incorrect or no answer; 1 = partially correct; 2 = correct and complete. The disagreements were resolved by consensus. Self-assessed knowledge, and opinion about the courses' usefulness and assessments/examinations were collected by nine questions on five-point Likert-type scales. Two additional questions collected the overall opinion of each course, as integer marks between 1 and 10. A Delphi technique was applied for assuring the face validity of this section, as well.

The ongoing EM course heavily employed information technology and used the video 360-degree technology as on-line substitutes for the practical classes. Students' opinion was collected with five questions on five-point Likert-type scales. Similarly to the gathered feed-back on previous courses, additional questions collected the students' overall opinion of activities related to this ongoing course (separately for lectures and practical classes, as integer marks between 1 and 10 for each) and a Delphi technique was applied for the questions' face validity.

Four questions collected students' opinions of the prospective employment of on-line educational activities in medical education (on five-point Likert-type scales) and their preference for future lectures.

The PHQ-9 questions were all required. The other questions were optional (except for the informed consent). For each item-group, the reliability of responses for this specific sample of participants was assessed by the internal consistency estimated using the Cronbach’s alpha coefficient.

Each section of the questionnaire also included open-ended questions. This paper is focused on the quantitative data, namely the answers to the close-ended questions from the project.

QUESTIONNAIRE

Analysis of the sustainability and resilience of data science knowledge and skills among the students at the University of Medicine and Pharmacy "Victor Babes" in Timisoara

Thank you for taking part in this survey.

You have been selected because you are a student at the University of Medicine and Pharmacy "Victor Babes" in Timisoara, enrolled in the general medicine program, the fourth year of study.

Replies are anonymous, no e-mail address or other personal information is recorded. Participation is entirely voluntary. You can always withdraw from this survey with no risk. By completing the questionnaire, you agree to the processing of the data collected.

If you have any questions about your participation in this research, you can contact us at one of the following addresses:

Alina Petrica: alina.petrica@urgentatm.ro
Diana Lungeanu: dlungeanu@umft.ro

* Required

Section 1 of 7

***Q1.** I confirm that I read and I understood the above information and the conditions in which this study is done, all the supplementary questions have received an answer and I agree to participate.
Yes

Section 2 of 7

Distance learning in medical education

Q2. Overall, how satisfied are you with the on-line learning experience?
1 to 5
1 = definitely unsatisfied
5 = very satisfied

Q3. Overall, how satisfied are you with your work on on-line learning platforms?
1 to 5
1 = definitely unsatisfied
5 = very satisfied

Q4. How satisfied are you with the on-line learning experience in March-July 2020?
1 to 5
1 = definitely unsatisfied
5 = very satisfied

Q5. To what extent do you appreciate the activities in March-July 2020 as contributing to your professional education?

1 to 5
1 = nothing at all
5 = very much

Q6. What was the percentage of teaching activities you attended in March-July 2020?

4: 75-100%
3: 74-50%
2: 49-25%
1: under 25%
0

Q7. What is the percentage of teaching activities you attended this semester?

4: 75-100%
3: 74-50%
2: 49-25%
1: under 25%
0

Q8. Overall, how satisfied are you with your life in this period? (Give a mark)

1 to 10 (mark)

Q9. In general, how do you appreciate your health? (Give a mark)

1 to 10 (mark)

Q10. How do you appreciate the support of the University in this period? (Give a mark)

1 to 10 (mark)

Section 3 of 7

PHQ-9 Patient Health questionnaire

Brief Deperession Severity Measure

Over the last 2 weeks, how often have you been bothered by any of the following problems?

For Q11 to Q19

1. Not at all
2. Several days
3. More than half the days
4. Nearly every day

***Q11**. Little interest or pleasure in doing things

***Q12**. Feeling down, depressed or hopeless

***Q13.** Trouble falling or staying asleep, or sleeping too much

***Q14.** Feeling tired or having little energy

***Q15.** Poor appetite or overeating

***Q16.** Feeling bad about yourself – or that you are a failure or have let yourself or your family down

***Q17.** Trouble concentrating on things, such as reading newspaper or watching TV

***Q18.** Moving or speaking so slowly that other people could have noticed? Or the opposite – being so fidgety or restless that you have been moving around a lot more than usual

***Q19.** Thoughts that you would be better off dead or of hurting yourself in some way

***Q20**. If you checked off *any* problems, how *difficult* have these problems made it for you to do your work, take care of things at home, or get along with other people?

1. Not difficult at all
2. Somewhat difficult
3. Very difficult
4. Extremely difficult

Section 4 of 7

ICT and biomedical data processing

In the following, when options are marked with → check all that apply
In the following, when options are marked with o → check only one

Q21. Which of the following are NOT operating systems:

Microsoft Edge
Android
Unix
iOS
Microsft Windows

Q22. Which of the following software applications can be used to create a medical data set:

Microsoft Excel
Microsoft Access
Epi Info
Microsoft Word
Google Sheets

Q23. A web address has the text 'https' next to the symbol of a padlock. What possible interpretations are valid:

The web page is locked
The web page is unsecure
The web page is secure
The web page is for shopping
The web page requires access key

Q24. Which of the following examples refer to on-line storage:

Moving some files from the hard-disk into Google Drive
Research using Wikipedia articles and subsequent links
On-line paying by credit card
Moving some files from the hard-disk into the Flash drive
Creating a new spreadsheet file in Google Drive

Q25. General Data Protection Regulation (GDPR) addresses:

Data protection and life privacy in the EU and EEA
Data storage and processing regarding the on-line social networks activity
Data processing by national military authorities
Data storage and processing regarding visa applications to a foreign state
Personal data export outside EU

Q26. You have a file with TXT format/extension. Which of the following are applications to visualize its content:

OpenOffice Writer
Notepad
Wordpad
Adobe Acrobat Reader DC
Microsft Work

Q27. Assume that you are using a DBMS application to create a medical database. The age of a patient will NOT be entered in a field/variable of type:

Character
Binary
Number
Nominal
Quantitative

Q28. You have a spreadsheet file with data from a questionnaire to which 300 people replied. The graph of choice to illustrate the answers to the question "What is your preferred holiday season?" is:

- Histogram
- Pie chart
- Bar chart
- Scatter chart
- Line chart

Q29. For the diagram below, the threshold value is moved to the right.

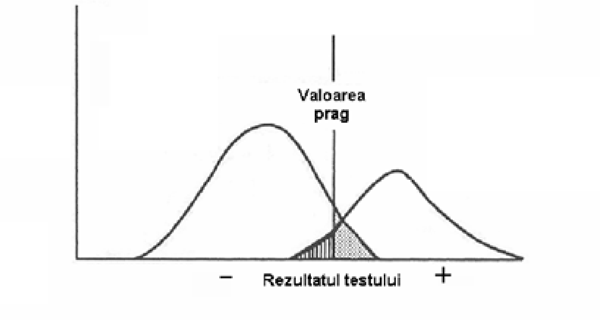

Which of the following is NOT true:

- False-positive rate decreases
- Specificity increases
- Sensitivity decreases
- Predictive values do not change
- Negative predictive value decreases

Q30. A statistical test fails to reject the hypothesis that there is a zero correlation between arterial pressure and cholesterol level. The test was applied with a level of significance of 5%. What is the correct interpretation of this result:

- It is improbable that observed correlation was by chance
- Observed results are not statistically significant; that is to say that there is not enough evidence to support the claim of an association between arterial pressure and cholesterol level
- Increased values of arterial pressure tend to associate with higer values of cholesterol
- Correlation between arterial pressure and cholesterol level is statistically significant ($p<0.05$)
- In the population from which the research sample was selected, correlation between arterial pressure and cholesterol level is zero

Q31. In a cross-sectional study, Odds Ratio (OR) equals 2.43; OR is the ratio between the outcome Odds in the exposed group and the outcome Odds in the non-exposed group. The conclusion is:

- OR signals a risk factor
- OR signals a protection factor
- Relative risk must also be calculated for a final conclusion
- In this type of study, OR cannot be calculated
- Incidence rate of the outcome must also be calculated for a final conclusion

Q32. Regarding a sample, only one of the following statements is true:

- It is a subgroup of the population of interest, on which statistical analysis is conducted
- Comprises all the individual with the same characteristics
- In general, it comprises more individuals than population of interest
- Irrespective of the selection criteria, the mean age in the sample must be identical to the population mean age
- Irrespective of the selection criteria, it has to be homogeneous in regard to the age

Q33. We consider the following series of numbers: 2; 2; 2; 4; 6; 12.
If number 14 is added to the series, then:

- Mean does not change
- Median does not change
- Mode does not change
- Mode increases
- Mean decreases

Q34. Concerning statistical data processing on samples of patients,
all the following are true, except for:

- The more individuals in a sample, the higher is the statistical power
- The more individuals in a sample, the higher is the statistical significance
- The fewer individuals in a sample, the higher is the chance for incorrect conclusions
- The more individuals in a sample, the higher is the chance for correct conclusions
- The more individuals in a sample, the weaker is the statistical significance

Section 5 of 7

Feed-back about the previous courses of data science

When answering the questions below, please think/refer to the courses of biomedical informatics (year 1) and biostatistics (year 2).

Q35. Using 'web-based' tools - I learnt:
1 to 5
1 = very little
5 = very much

Q36. Data management – I learnt:
1 to 5
1 = very little
5 = very much

Q37. Description and data visualisation – I learnt:
1 to 5
1 = very little
5 = very much

Q38. Data analysis – I learnt:
1 to 5
1 = very little
5 = very much

Q39. Synthesis and interpretation of statistical results - I learnt:
1 to 5
1 = very little
5 = very much

Q40. The evaluation of knowledge was:
1 to 5
1 = completely innapropriate
5 = very appropriate

Q41. The evaluation of practical competencies and skills was:
1 to 5
1 = completely innapropriate
5 = very appropriate

Q42. The courses have proved useful in the years 2-4 of the medical programe:
1 to 5
1 = definitely not
5 = completely agree

Q43. The courses proved useful between March and November 2020:
1 to 5
1 = definitely not
5 = completely agree

Q44. Overall, how satisfied are you with the course of biomedical informatics? (Give a mark)
1 to 10 (mark)

Q45. Overall, how satisfied are you with the course of biostatistics ? (Give a mark)
1 to 10 (mark)

Q46. What do you think it should be done to improve the courses? (open question)
____

Section 6 of 7

Emergency Medicine

When answering the questions below, please think/refer to the ongoing course of emergency medicine (year 4).

Q47. In this course– I learnt about:

Q47.1. Cardio-pulmonary resuscitation
1 to 5
1 = nothing
5 = very much

Q47.2. Management of the traumatised patient
1 to 5
1 = nothing
5 = very much

Q47.3. Management of the patient with shock
1 to 5
1 = nothing
5 = very much

Q48. E-learning trainings can supplement/replace the practical classes or training:
1 to 5
1 = definitely not
5 = completely

Q49. Practical knowledge/abilities can be assessed on-line:
1 to 5
1 = definitely not
5 = completely

Q50. To improve on-line classes, I think ... (open question)

Q51. Overall, how satisfied are you with the practical trainings of this course? (Give a mark)
1 to 10 (mark)

Q52. Overall, how satisfied are you with the lectures of this course? (Give a mark)
1 to 10 (mark)

Q53. I am familiar with video 360:
1 to 5
1 = definitely not
5 = very much

Q54. Filmed scenarios 360 have an added value to the emergency medicine knowledge:
1 to 5
1 = definitely not
5 = very much, totally agree

Section 7 of 7

In the end.......

Q55. I have the ICT competencies and skills to independently participate in e-learning:
1 to 5
1 = definitely not
5 = completely agree

Q56. I am more motivated to attend e-learning classess than I was in the traditional education:
1 to 5
1 = definitely not
5 = completely agree

Q57. When the COVID-19 pandemic restrictions cease, e-learning will be a suitable complement to traditional activities in medical education
1 to 5
1 = definitely not
5 = completely agree

Q58. Considering the present e-learning experience, for future lectures and theoretical presentations, I prefer:
- Traditional face-to-face lectures
- e-Learning
- A mixture of the two above

Q59. To improve e-learning activities in medical education, I think ... (open question)

____

Q60. To compensate for the lack of practical activities during this period, I think ... (open question)

____

Q61. My gender ...
- F
- M
- I prefer not to answer

Q62. My age (years) ________
