# Supplementary material for: Beyond the Digital Competencies of Medical Students: Concerns over Integrating Data Science Basics into the Medical Curriculum
Source: Int J Environ Res Public Health. 2022 Nov 30;19(23):15958. doi: 10.3390/ijerph192315958 (PMC9739359; doi:10.3390/ijerph192315958)
Supplement: Supplementary file 1 [file ijerph-19-15958-s001.zip › ICTbioStats-SupplementaryFile3.pdf]

Table S1. Declared on-line attendance, students' overall satisfaction with life and on-line professional activity, and the PHQ-9 total scoring. Data were separately gathered as a recollection of the spring in 2020 (during the pandemic outbreak) and in regard to the ongoing first semester of the academic year 2020-2021. The improvement on all aspects was significant during the latter period, associated with more stability in life in general. The contribution of personal commitment to attend the academic activities was also significant (there was a statistically significant linear association between the attendance levels in the two periods, both with exclusively on-line activities). The Cronbach' alpha value for the PHQ-9 reflects good reliability.

Question/ Variable		All N=121	Males N=28	Females N=84	Not declared N=9	p-value (a),(b),(c),(d),(e)
Q62	Age[(a)]	22.17 ± 0.843	22.25 ± 0.645	22.15 ± 0.938	22.11 ± 0.333	0.854
Q2	On line education[(b)]	3 (2 – 4)	3 (3 – 4)	3 (2 – 4)	3 (2 – 3)	0.298
Q3	On-line work satisfaction[(b)]	3 (2 – 4)	3 (2 – 3.5)	3 (2 – 4)	2 (2 – 3)	0.185
Q4	Experience in spring 2020[(b)]	2 (2 – 3)	2.5 (2 – 3.5)	2 (2 – 3)	2 (1 – 3)	0.212
Q5	Professionally prepared in spring 2020[(b)]	2 (2 – 3)	2 (2 – 3)	2 (2 – 3)	2 (1 – 3)	0.659
	Q2 vs. Q4; Wilcoxon signed rank test, p < 0.001**					
	Q3 vs. Q5; Wilcoxon signed rank test, p < 0.001**					
Q6	Attendance spring 2020[(c)]					
	less than 25%	14 (11.6%)	1 (3.6%)	12 (14.3%)	1 (11.1%)	0.795
	49-25%	18 (14.9%)	5 (17.6%)	11 (13.1%)	2 (22.2%)	
	74-50%	22 (18.2%)	5 (17.6%)	16 (19%)	1 (11.1%)	
	75-100%	67 (55.4%)	17 (60.7%)	45 (53.6%)	5 (55.6%)	
Q7	On-line attendance current semester[(c)]					
	less than 25%	2 (1.7%)	–	1 (1.2%)	1 (11.1%)	0.123
	49-25%	5 (4.1%)	1 (3.6%)	3 (3.6%)	1 (11.1%)	
	74-50%	13 (10.7%)	1 (3.6%)	10 (11.9%)	2 (22.2%)	
	75-100%	101 (83.5%)	26 (92.9%)	70 (83.3%)	5 (55.6%)	
	Q6 vs. Q7; statistical test for linear association, Monte-Carlo simulation (10000 sampled tables), p = 0.003**					
Q8	Life satisfaction[(d)]	7 (4.5 – 8)	7 (6 – 8.5)	7 (4 – 8)	6 (3 – 6)	0.029*
Q9	Health satisfaction[(d)]	9 (8 – 9.5)	9 (8 – 10)	9 (8 – 9)	8 (6 – 9)	0.284
Q10	Perceived support from University[(d),#]	7 (4 – 8) [#]	6 (2.5 – 8) [#]	7 (4 – 8)	5 (3 – 7)	0.594
Q11 to Q20	PHQ-9 total27[(e)]	8 (5 – 11)	7.5 (4.5 – 10.5)	8 (5 – 11)	9 (6 – 11)	0.604
	9 items, Cronbach's alpha = 0.885					

[(a)] mean ± std dev; ANOVA statistical test for significance of observed differences between the three-sex groups

[(b)] rank scores between 1 and 5; median (IQR); Kruskal-Wallis statistical test for significance of observed differences between the three-sex groups

[(c)] counts (%); Chi-square statistical test for significance of observed differences between the three-sex groups (either asymptotic, or Monte-Carlo simulation based on 10000 samples)

[(d)] mark between 1 and 10; median (IQR); Kruskal-Wallis statistical test for significance of observed differences between the three-sex groups

[(e)] median (IQR); Kruskal-Wallis statistical test for significance of observed differences between the three-sex groups

Notations: IQR inter-quartile range; std dev, standard deviation; PHQ-9, nine-item Patient Health Questionnaire

[#] one missing value for a male respondent

*,** statistical significance, p < 0.05, p < 0.01

Table S2. Self-assessed ICT abilities for independent on-line activities; and opinions of the prospective applicability of e-learning and on-line activities in medical education.

Question/ Variable		All N=121	Males N=28	Females N=84	Not declared N=9	p-value [(a),(b)]
Q55	ICT abilities for on-line activities[(a)]	4 (3 – 5)	3 (4 – 5)	3 (4 – 5)	3 (3 – 5)	0.866
Q56	Motivated to attend[(a)]	3 (1 – 4)	3 (2 – 4)	3 (1 – 4)	2 (1 – 3)	0.206
Q57	On-line activities are a valid complement to face-to-face[(a)]	3 (2 – 4)	3 (2 – 4)	3 (1 – 4)	3 (2 – 4)	0.898
Q58	Preferred lectures in the future[(b)]					
	no preference	2 (1.7%)	–	2 (2.4%)	–	0.885
	face-to-face classical teaching	50 (41.3%)	10 (35.7%)	35 (41.7%)	5 (55.6%)	
	eLearning	13 (10.7%)	4 (14.3%)	8 (9.5%)	1 (11.1%)	
	combination	56 (46.3%)	14 (50%)	39 (46.4%)	3 (33.3%)	

[(a)] rank scores between 1 and 5; median (IQR); Kruskal-Wallis statistical test for significance of observed differences between the three-sex groups

[(b)] counts (%); Chi-square statistical test for significance of observed differences between the three-sex groups (either asymptotic, or Monte-Carlo simulation based on 10000 samples)

Notation: IQR inter-quartile range

Table S3. Two-by-two correlation matrix between the PHQ-9 scoring, satisfaction with on-line education, life, health and perceived support from University, and the overall marks students gave to the data science courses.

Variable		PHQ-9 total	On line education(	On-line work satisfaction	Life satisfaction	Health satisfaction	Perceived support from University#	Usefulness BMI & Biostats	mark BMI	mark Biostats
PHQ-9 total	R	1.000	-.248**	-.314**	-.395**	-.433**	-.303**	-.260**	-.241**	-.270**
	p	.	0.006	<0.001	<0.001	<0.001	0.001	0.004	0.008	0.003
	N	121	121	121	121	121	120	121	121	121
On line education(	R	-.248**	1.000	**.686****	**.521****	.345**	**.612****	.409**	.444**	.327**
	p	0.006	.	<0.001	<0.001	<0.001	<0.001	<0.001	<0.001	<0.001
	N	121	121	121	121	121	120	121	121	121
On-line work satisfaction	R	-.314**	.686**	1.000	.448**	.274**	.488**	.364**	.306**	.209*
	p	<0.001	<0.001	.	<0.001	0.002	<0.001	<0.001	0.001	0.021
	N	121	121	121	121	121	120	121	121	121
Life satisfaction	R	-.395**	.521**	.448**	1.000	.403**	.349**	.260**	.360**	.293**
	p	<0.001	<0.001	<0.001	.	<0.001	<0.001	0.004	. <0.001	0.001
	N	121	121	121	121	121	120	121	121	121
Health satisfaction	R	-.433**	.345**	.274**	.403**	1.000	.457**	.277**	.333**	.346**
	p	<0.001	<0.001	0.002	<0.001	.	<0.001	0.002	<0.001	<0.001
	N	121	121	121	121	121	120	121	121	121
Perceived support from University#	R	-.303**	.612**	.488**	.349**	.457**	1.000	.487**	**.531****	.409**
	p	0.001	<0.001	<0.001	<0.001	<0.001	.	<0.001	<0.001	<0.001
	N	120	120	120	120	120	120	120	120	120
Usefulness BMI & Biostats	R	-.260**	.409**	.364**	.260**	.277**	.487**	1.000	**.591****	**.581****
	p	0.004	<0.001	<0.001	0.004	0.002	. <0.001	.	<0.001	<0.001
	N	121	121	121	121	121	120	121	121	121
mark BMI	R	-.241**	.444**	.306**	.360**	.333**	.531**	.591**	1.000	**.869****
	p	0.008	<0.001	0.001	<0.001	<0.001	<0.001	<0.001	.	<0.001
	N	121	121	121	121	121	120	121	121	121

Variable		PHQ-9 total	On line education(	On-line work satisfaction	Life satisfaction	Health satisfaction	Perceived support from University#	Usefulness BMI & Biostats	mark BMI	mark Biostats
mark Biostats	R	-.270**	.327**	.209*	.293**	.346**	.409**	.581**	.869**	1.000
	p	0.003	<0.001	0.021	0.001	<0.001	<0.001	<0.001	<0.001	.
	N	121	121	121	121	121	120	121	121	121

Notations: BMI, biomedical informatics; N, number of paired values in the correlation analysis; p, p-value for statistical significance; R, Spearman coefficient of correlation. Significant R values over 0.5 are in bold.

one missing value for a male respondent

Table S4. Two-by-two correlation matrix between the PHQ-9 scoring, satisfaction with on-line education, life, health and perceived support from University, and the overall marks students gave to the EM course.

Variable		PHQ-9 total	On line education(	On-line work satisfaction	Life satisfaction	Health satisfaction	Perceived support from University#	mark EM practicals	mark EM lectures
PHQ-9 total	R	1.000	-.248**	-.314**	-.395**	-.433**	-.303**	-.144	-.186*
	p	.	0.006	<0.001	<0.001	<0.001	0.001	0.115	0.041
	N	121	121	121	121	121	120	121	121
On line education(	R	-.248**	1.000	**.686****	**.521****	.345**	**.612****	.440**	.282**
	p	0.006	.	<0.001	<0.001	<0.001	<0.001	<0.001	0.002
	N	121	121	121	121	121	120	121	121
On-line work satisfaction	R	-.314**	.686**	1.000	.448**	.274**	.488**	.336**	.279**
	p	<0.001	<0.001	.	<0.001	0.002	<0.001	<0.001	0.002
	N	121	121	121	121	121	120	121	121
Life satisfaction	R	-.395**	.521**	.448**	1.000	.403**	.349**	.307**	.248**
	p	<0.001	<0.001	<0.001	.	<0.001	<0.001	0.001	0.006
	N	121	121	121	121	121	120	121	121
Health satisfaction	R	-.433**	.345**	.274**	.403**	1.000	.457**	.421**	.297**
	p	<0.001	<0.001	0.002	<0.001	.	<0.001	<0.001	0.001
	N	121	121	121	121	121	120	121	121
Perceived support from University#	R	-.303**	.612**	.488**	.349**	.457**	1.000	.473**	.449**
	p	0.001	<0.001	<0.001	<0.001	<0.001	.	<0.001	<0.001
	N	120	120	120	120	120	120	120	120
mark EM practicals	R	-.144	.440**	.336**	.307**	.421**	.473**	1.000	**.730****
	p	0.115	<0.001	<0.001	0.001	<0.001	<0.001	.	<0.001
	N	121	121	121	121	121	120	121	121
mark EM lectures	R	-.186*	.282**	.279**	.248**	.297**	.449**	**.730****	1.000
	p	0.041	0.002	0.002	0.006	0.001	<0.001	<0.001	.
	N	121	121	121	121	121	120	121	121

Notations: EM, emergency medicine; N, number of paired values in the correlation analysis; p, p-value for statistical significance; R, Spearman coefficient of correlation. Significant R values over 0.5 are in bold.

one missing value for a male respondent
